# Supplementary figures and images for: Pyruvate carboxylase supports the pulmonary tropism of metastatic breast cancer
Source: Breast Cancer Res. 2018 Jul 13;20:76. doi: 10.1186/s13058-018-1008-9 (PMC6045837; doi:10.1186/s13058-018-1008-9)

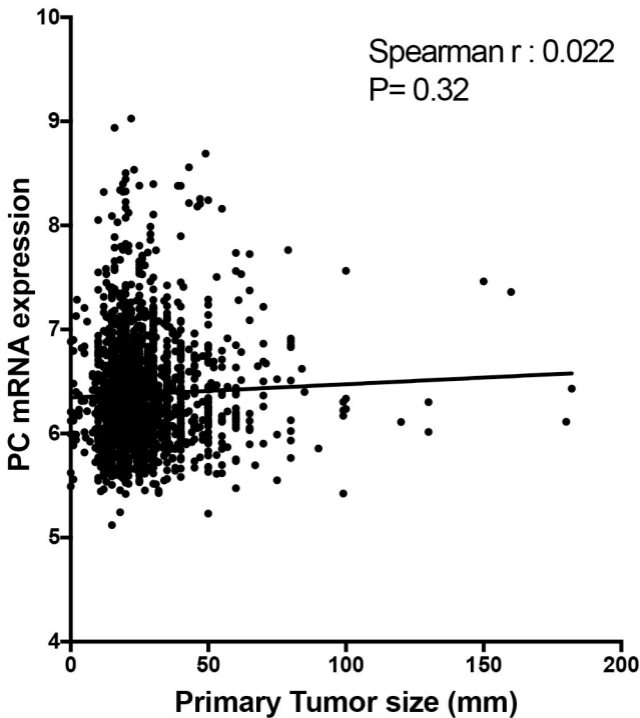

Supplement: Supplementary file 1 — Figure S1. PC expression is not correlated with primary tumor size. Patient samples within the METABRIC dataset were analyzed for PC expression in relation to primary tumor size. Data are analyzed by a nonparametric Spearman correlation resulting in the indicated r and P values. The linear regression for these data is also shown. (PDF 235 kb) [file 13058_2018_1008_MOESM1_ESM.pdf]

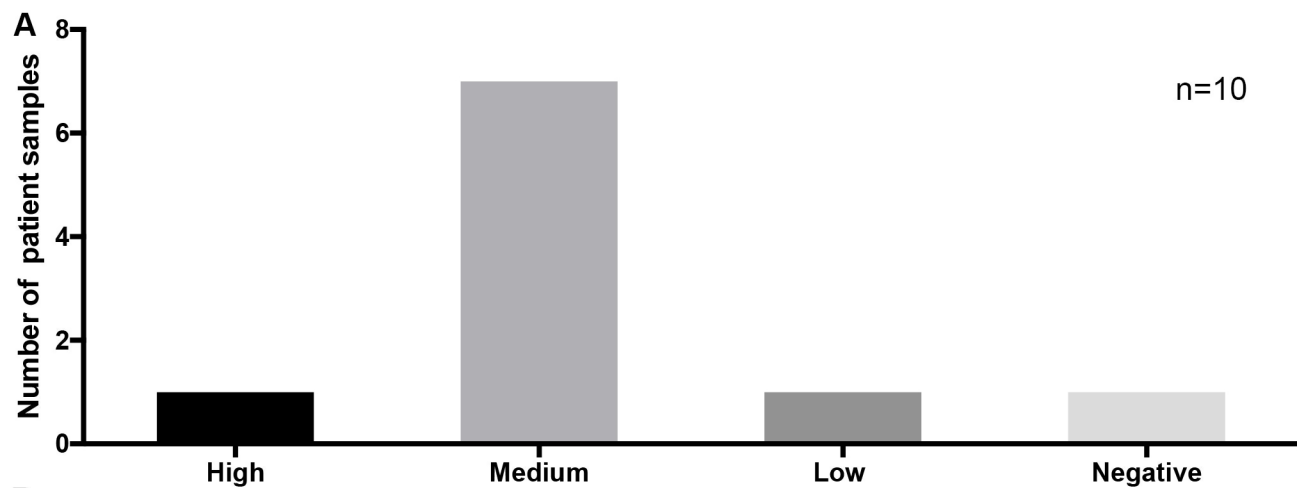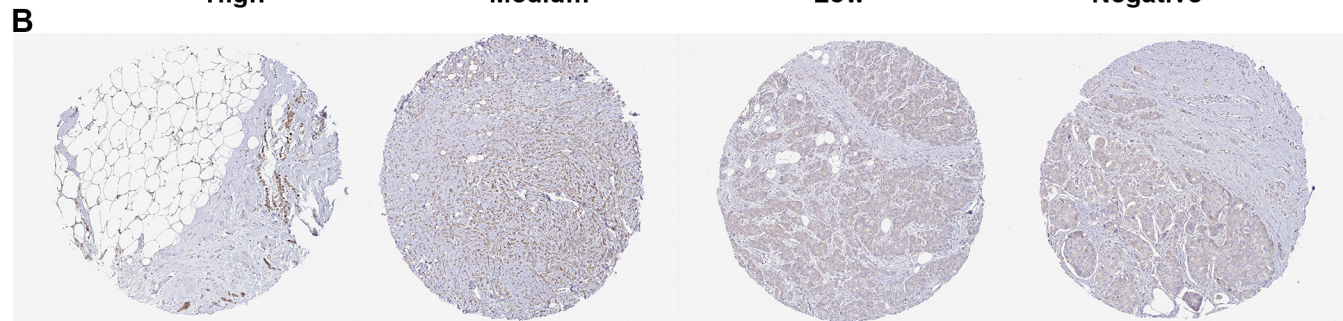

Supplement: Supplementary file 2 — Figure S2. PC expression in primary breast tumors. PC expression within tumor cells was rated as high, medium, low, or negative (n = 10). Representative sections from each group are shown. Data was obtained from the protein atlas dataset (www. proteinatlas.org) [33]. (PDF 2805 kb) [file 13058_2018_1008_MOESM2_ESM.pdf]

**A****TGF- $\beta$ 1**

4 days

**-****+**4T1  
scram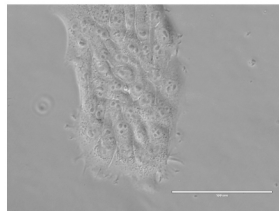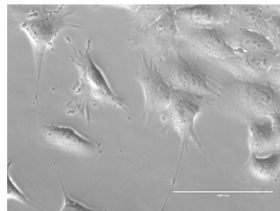4T1  
shPC  
25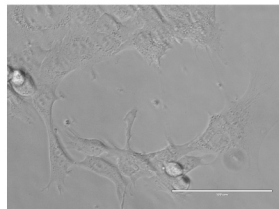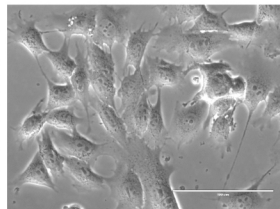4T1  
shPC  
28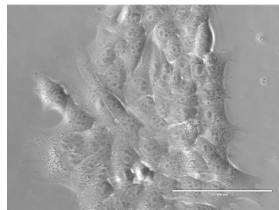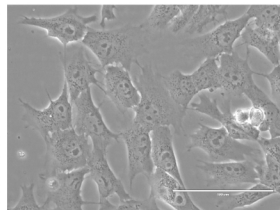**B**

4T1-scram

4T1-shPC 25

4T1-shPC 28

-

+

-

+

-

+

PC

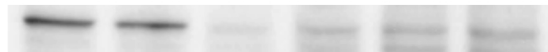 $\beta$ -tub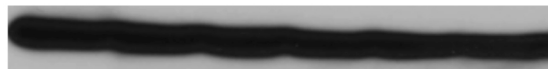**C**

Dox ng/ml

0

100

500

750

1000

Snail

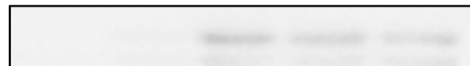

PC

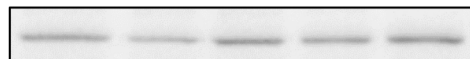 $\beta$ -Tub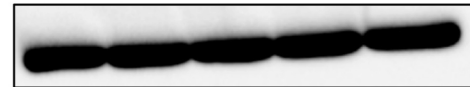

Supplement: Supplementary file 3 — Figure S3. Transient induction of EMT does not induce expression of PC. (A) Photomicrographs showing EMT-like changes in cellular morphology of the control (scram) and PC-depleted (shPC25 and shPC28) 4 T1 cells upon treatment with exogenous TGF-β1 (5 ng/ml) for 4 days. (B) Immunoblot analyses for PC in cells shown in Panel A. β-tubulin served as a loading control. (C) The RAS transformed MCF-10A-T1 k cells were constructed to express a doxycycline-inducible vector encoding the EMT transcription factor Snail. Expression of PC was analyzed upon addition of doxycycline (Dox) at the indicated concentrations. Expression of Snail and β-tubulin (β-Tub) served as loading controls. (PDF 1401 kb) [file 13058_2018_1008_MOESM3_ESM.pdf]

**A**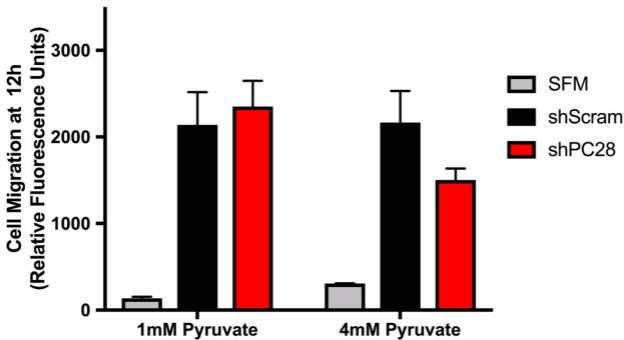**B**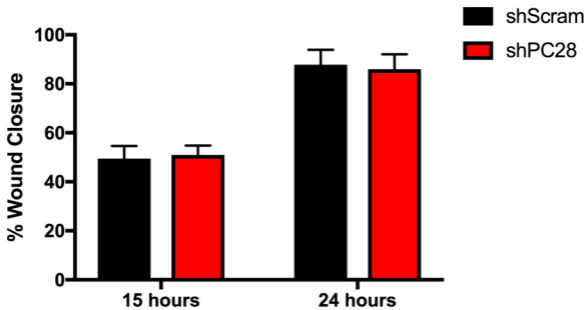

Supplement: Supplementary file 4 — Figure S4. PC is not required for cell migration. (A) Control (shScram) and PC depleted (shPC28) cells were plated into 8 μm transwell inserts in serum-free media containing 1 or 4 mM pyruvate. Cell migration was quantified 12 h after plating using calcein AM. SFM represents wells where the bottom chamber was filled with serum-free media as negative control. Values are presented as mean relative fluorescence units, ±SEM. (B) Control (shScram) and PC-depleted (shPC28) 4 T1 monolayers were wounded and closure was measured 15 and 24 h later. Values are presented as mean percent would closure, ±SEM. (PDF 372 kb) [file 13058_2018_1008_MOESM4_ESM.pdf]

**4T1**

---

**Primary tumor**

**Lung Metastasis**

**HIF1- $\alpha$**

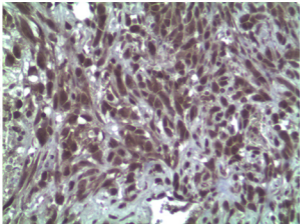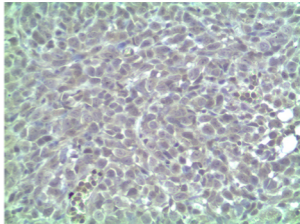

Supplement: Supplementary file 5 — Figure S5. HIF-1α expression that characterizes primary tumors is lost in pulmonary metastases. 4 T1 cells were engrafted onto the mammary fat pad via an intraductal injection and grown as primary tumors. These tumors gave rise to spontaneous pulmonary metastases. Upon necropsy both primary and metastatic tumors were analyzed by immunohistochemistry for the expression of HIF-1α. Nuclear expression of HIF-1α is very high in viable primary tumor tissue. In contrast, nuclear HIF-1α is drastically reduced in pulmonary metastases. Data are representative of three separate mice bearing primary tumors and metastases. (PDF 1389 kb) [file 13058_2018_1008_MOESM5_ESM.pdf]
